# Are social conflicts at work associated with depressive symptomatology? Results from the population-based LIFE-Adult-Study

**DOI:** 10.1186/s12995-020-0253-x

**Published:** 2020-02-12

**Authors:** Andrea E. Zuelke, Susanne Roehr, Matthias L. Schroeter, A. Veronica Witte, Andreas Hinz, Christoph Engel, Cornelia Enzenbach, Joachim Thiery, Markus Loeffler, Arno Villringer, Steffi G. Riedel-Heller

**Affiliations:** 1 Institute of Social Medicine, Occupational Health and Public Health (ISAP), Medical Faculty, University of Leipzig, Philipp-Rosenthal-Str. 55, 04103 Leipzig, Germany; 2 Max Planck Institute for Human Cognitive and Brain Sciences, Leipzig, Germany; 3 University Hospital Leipzig, Day Clinic for Cognitive Neurology, Leipzig, Germany; 4 Department of Medical Psychology and Medical Sociology, University of Leipzig, Leipzig, Germany; 5 Institute for Medical Informatics, Statistics and Epidemiology (IMISE), University of Leipzig, Leipzig, Germany; 6 Institute of Laboratory Medicine, Clinical Chemistry and Molecular Diagnostics (ILM), University Hospital Leipzig, Leipzig, Germany

**Keywords:** CES-D, Depressive symptoms, Mental health, Psychosocial work environment, Social conflict, Multilevel model, O*NET

## Abstract

**Background:**

Psychosocial stressors in the workplace can be detrimental to mental health. Conflicts at work, e.g. aggression, hostility or threats from coworkers, supervisors or customers, can be considered a psychosocial stressor, possibly increasing risk for depressive symptoms. Existing studies, however, differ in the assessment of social conflicts, i.e. as individual- or job-level characteristics. Here, we investigated the association between conflicts at work assessed as objective job characteristics, and depressive symptomatology, using data from a large population-based sample. Additionally, we investigated gender differences and the impact of personality traits and social resources.

**Methods:**

We used data from the population-based LIFE-Adult-Study from Leipzig, Germany. Information on conflicts at work, assessed as job characteristics, were drawn from the Occupational Information Network, depressive symptoms were assessed via the Center for Epidemiological Studies Depression Scale. Multilevel linear regression models with individuals and occupations as levels of analysis were applied to investigate the association between conflicts at work and depressive symptoms.

**Results:**

Our sample included 2164 employed adults (age: 18–65 years, mean: 49.3, SD: 7.9) in 65 occupations. No association between conflicts s at work and depressive symptomatology was found (men: b = − 0.14; *p* = 0.74, women: b = 0.17, *p* = 0.72). Risk for depression was mostly explained by individual-level factors like e.g. neuroticism or level of social resources. The model showed slightly higher explanatory power in the female subsample.

**Conclusion:**

Conflicts at work, assessed as objective job characteristics, were not associated with depressive symptoms. Possible links between interpersonal conflict and impaired mental health might rather be explained by subjective perceptions of social stressors and individual coping styles.

## Background

The working environment and job characteristics have a crucial influence on well-being and mental health, which in turn impact work performance and productivity [1–3]. Given the average duration of working life in the European Union currently amounts to 36.2 years, meaning that people spend more than one third of their lives in employment, it can be assumed that many sources of perceived stress are encountered in the workplace [4, 5]. Therefore, understanding how the social environment at work can enhance or impair mental health is crucial. Occupational health research in the last decades has particularly focused on the dimensions of work demands and control, drawing on Karasek and Theorell’s demand-control-model [2, 6–8]. Later amendments have added another dimension – social support –, leading to the demand-control-support-model [9, 10]. It is assumed that impaired mental health and well-being can be found especially in people holding jobs characterized by high strain (high demands but low control) and low social support. Recent meta-analyses found higher risks for clinical depression [2, 7, 11] and depressive symptoms [6] in people experiencing job strain, reporting mostly small to medium effect sizes.

While the demand-control-(support) model provides a highly valuable measure for important aspects of work organization, it has been argued that other psychosocial aspects of the working environment are still understudied [6, 12–15]. This also applies to social conflicts at work and their possible association with mental health [16, 17]. The World Health Organization emphasizes the meaning of interpersonal relations at work for (mental) health, stressing that poor relations and conflicts with co-workers or supervisors can increase risk for mental illness [18]. Interestingly, the original article introducing the demand-control-model explicitly named social conflicts at work as a stressor, stating that job demands include “psychological stressors involved in accomplishing the workload, stressors relating to unexpected tasks, and stressors of job-related personal conflict” [8]. Against this background, social conflicts can be considered a stressful work demand, increasing risk of depression.

Social conflicts in the workplace can broadly be defined as a range of interpersonal maltreatment behaviors. It can include e.g. rude behavior, verbal aggression, bullying or physical assault [16, 19] and can result in negative consequences such as increased rates of turnover, less productivity and employee satisfaction [20, 21]. The literature supports a link between higher rates of interpersonal conflict at work and depression, whereas interpersonal conflict acts as a major stressor. Most investigations of social conflicts at work focus on occupations in the service sector, e.g. cashiers, call-center agents or bus drivers, since these jobs entail a high level of interpersonal contacts with colleagues and customers [22]. Somewhat paradoxically, the literature also reports a high prevalence of social conflict in professions with a strong focus on caring like nursing [23, 24] or teaching [25, 26]. Several studies in different work settings reported social conflict at work the single most important source of perceived stress [17, 27, 28].

A common criticism concerning studies on occupational mental health refers to the fact that most empirical studies rely on self-reported measures both of independent and outcome variables [6, 29–34]. Therefore, individuals in identical jobs can rate the amount of job stress or, specifically, work-related conflict quite differently. This might especially be true for people experiencing depressive symptoms, which may influence their affective assessment of their job and working environment [32]. Furthermore, only few validated instruments measuring subjective job-related stressors are available. To account for this risk of bias, a growing body of research investigates associations between psychosocial factors of working environments and mental health by drawing on objective assessments of occupational information, e.g. [30, 35–37]. Rather than broad categories like “perceived stress”, these assessments could possibly provide a clearer indication of the actual environmental conditions that are linked to depression and, therefore, knowledge on what aspects of the working environment need changing, allowing for effective prevention strategies [38, 39]. Lastly, previous studies on occupational mental health have often focused on jobs in the service sector, using rather small and very specific samples. More comprehensive investigations using population-based samples are currently rare, especially in Germany.

Another point of discussion refers to the level and unit of analysis: It can be argued that occupational stressors refer to qualities of jobs rather than of individual people [29, 40–42]. Despite this, most empirical investigations so far have relied solely on individual-level data. That said, workers holding the same job, i.e. individuals nested within jobs, cannot reasonably be regarded as independent units of analysis, which in turn violates important assumptions of standard ordinary least squares (OLS) regression techniques [29]. Ignoring the potential effect of clustering bears the risk of over-estimating the importance of regression coefficients [40]. Therefore, it has been argued that the hierarchical structure of workers in jobs shall be recognized by choosing appropriate techniques of analysis like e.g. multilevel modeling [41, 42].

Several factors have been identified to moderate the link between psychosocial work characteristics and depressive symptoms. Neuroticism and extraversion have been found to be linked to depressive symptoms: There is a strong correlation between neuroticism and increased risk of depression, while high levels of extraversion act as a protective factor against depressive symptomatology [43–46]. It has been shown empirically that the negative effect of neuroticism is especially pronounced under stressful conditions, i.e. adversity and conflictual situations are especially harmful for people showing high degrees of neuroticism [47]. Social support from friends or family has been found a protective factor against depression in several studies (for an overview, see [48]). Protective effects against depression have also been found for higher levels of education [49, 50].

The role of gender in the relationship between work-related psychosocial stressors and mental health is still inconclusive. While some researchers report stronger effects of occupational stressors on men’s health [51], others found the relationship to be stronger in women [52, 53] or reported no gender differences [6]. Possible gender differences in the association of work-related stressors with depressive symptoms might occur for different reasons: First, women and men can differ in the degree of exposure to occupational stressors, namely: interpersonal conflict. While most studies reported men and women to be equally affected by conflicts in the workplace, others found higher rates of exposure among women (for an overview, see [54]). More detailed investigations revealed that men mostly reported conflicts with male supervisors, whereas women experience conflicts both with men and women alike and with both supervisors and colleagues in equal proportions [54]. Women, however, are more likely to perceive conflictual situations as sexual harassment [55], which might possibly overlap with social conflicts. Second, men and women might differ in their coping strategies, i.e. ways of handling interpersonal conflict, or in their resources available for handling stressors at work. It has been shown that, due to gendered socialization processes, men tend to cope with stress more instrumentally, while women are more likely to openly express emotions [17, 56]. Studies on (occupational) stress have found men to use more problem-oriented strategies whereas women are, on average, more emotion-focused [17].

Against this background, this study seeks to investigate the association between conflict at work and depressive symptoms, using a large population-based sample comprising a variety of different occupations. We hypothesize that a) higher levels of conflict at work are associated with increased depressive symptoms, b) the association will be smaller than in studies using self-report measures of interpersonal conflict. This is due to the objective assessment of interpersonal conflict as a feature of occupations in our study which does not capture different individual perceptions of stressors between workers holding the same job. We further investigate the influence of c) personality traits, i.e. neuroticism and extraversion, as well as social resources and education on the association. Neuroticism is assumed to be linked to increased levels of depression, whereas extraversion, higher levels of social resources and education should be associated with decreased depressive symptoms. Lastly, we seek to investigate possible gender differences.

## Subjects and methods

### Participants

We used data from the LIFE-Adult-Study, a population-based cohort study conducted by the Leipzig Research Center for Civilization Diseases. 10,000 randomly selected inhabitants of Leipzig, Germany (aged between 18 and 79 years) completed the baseline-examination between 2011 and 2014. The LIFE-Study aims to investigate the prevalence, genetic predispositions and modifiable lifestyle factors of major civilization diseases such as cardiovascular diseases, dementia or depression. Physical examinations, structured interviews and questionnaires were administered to all participants as part of the baseline assessment. Pregnancy and insufficient command of the German language were exclusion criteria. For a detailed description of the study aims and concept, see [57]. The study included an age- and sex-stratified random sample of 10,000 community-dwelling German-speaking residents of the city of Leipzig who were randomly drawn from lists provided by the local registry office. These residents were sent an invitation letter, containing information on the aims and design of the study, and a response form. If residents did not respond, a reminder invitation was sent. Non-responders were searched in public phone directories and contacted by phone. For residents who refused to participate, residents of the same age and sex were randomly drawn from the registry office’s lists and invited to participate.

Out of the initial study sample, we excluded cases aged 66 years and older (*n* = 3249 cases) in order to exclude individuals who had already retired. Additionally, individuals who were not working (*n* = 1446 cases), working less than 15 h per week (*n* = 159) or had no information on current employment status (*n* = 10) were dropped from the analyses. We further excluded cases with missing values on CES-D score (*n* = 348), LSNS (*n* = 217), NEO-16 AM-info on neuroticism/extraversion (*n* = 318), education (n = 1) and occupations that could not be clearly matched with an O*NET-occupation identifier (*n* = 42). To avoid bias caused by small groups, observations were dropped if the respective occupation had less than 10 incumbents in the dataset (*n* = 1096 observations). Finally, we excluded cases with missing values on the conflict-variables (*n* = 46 cases) and the lowest quintiles for the variables “frequency of conflict situations” (*n* = 427), “dealing with unpleasant or angry people” (*n* = 167), “dealing with physically aggressive people” (*n* = 310). The final sample contained 2164 individuals.

### Measures

#### Depressive symptoms

Depressive symptoms were assessed using the Center for Epidemiologic Studies Depression Scale (CES-D [58]). This self-report scale comprises 20 items, assessing depressive symptoms such as depressed mood, hopelessness or insecurity during the last week, using a 4-point-Likert-scale (0 = never/almost none of the time; 3 = most or all of the time). The score ranges from 0 to 60 points, with higher values indicating higher levels of current depressive symptomatology. Drawing on reference values from comparable population-based samples, a cut-off value of ≥23 points indicates risk of depression [59].

#### Individual-level covariates

We included gender and age as individual-level covariates in our analyses. To control for social resources, we used information from the short-form of the Lubben Social Network-Scale (LSNS-6), a measure assessing perceived social resources and support. Questions include e.g. “How many friends/relatives do you see or hear from at least once a month?” or “How many friends/relatives do you feel close to such as that you could call on them for help?”. Possible scores range from 0 to 30 points, higher scores indicating higher levels of social resources. A score below 12 points is considered an indicator of social isolation [60]. We further controlled for neuroticism and extraversion as assessed by the NEO-16 Adjective Measure [61]. Neuroticism and extraversion were assessed with four and three items, respectively. Participants rated themselves on a 7-point scale ranging from 1 (disagree strongly) to 7 (agree strongly), with the common introduction “I see myself as: (e.g. item 5: anxious)”. We classified education (low, middle, high) based on the CASMIN-scale (Comparative Analysis of Social Mobility in Industrialized Nations), which takes into account general and vocational education [62].

#### Occupational-level covariates

We used occupational information from the Occupational Information Network (O*NET) database (version 23.2). The O*NET-database was developed by the US Department of Labor/Employment and Training Administration (USDOL/ETA) and provides detailed information on a total of over 900 different occupations within the US-American labor-market [63]. Data are provided by job incumbents, supervisors and occupational experts. Comparable databases for the German labor market are currently not available. O*NET data have been used to measure associations of work-related factors with depressive symptoms [35], cardiovascular disease [30], clinical depression [36], self-rated health and hypertension [64], among others. For every type of occupation, a comprehensive set of descriptors is available, including information on required skills, knowledge, values and activities common in the respective occupation. Among the section on worker activities, items assessing the importance and level/frequency of several types of social interactions in the workplace are available. Interpersonal conflict is assessed with three items: *frequency of conflict situation; dealing with unpleasant or angry people; dealing with physically aggressive people*. The three items were combined into one additive “conflict-score” as an overall-measure of interpersonal conflict in the workplace by summing up the values of the three respective items. Cronbach’s alpha was 0.87, indicating a high level of internal consistency.

Since some occupations experience literally no conflictual contacts, observations were grouped into quintiles based on their respective scores in the three conflict-items. The lowest quintile was then removed from the analysis sample. To avoid the risk of bias due to statistical outliers, we excluded occupations with less than 10 incumbents from the sample.

### Statistical analyses

To describe the sample with regard to individual- and occupational-level characteristics, Chi^2^- and two-sample t-tests were used as appropriate. We conducted an overall-analysis of the complete sample as well as separate analyses for men and women. Subsequently, we investigated associations between interpersonal conflict in the workplace and depressive symptomatology by calculating a linear multilevel regression model. Multilevel models are suited for analyzing hierarchically structured data, e.g. individuals (level 1-units) clustered in occupations (level 2-units). *P*-values < 0.05 were considered significant, and all models were calculated using maximum likelihood estimation. Analyses were conducted using Stata (SE) 13.1. We first fit an empty model (null model), containing only the random effects of individuals and occupations, to determine the proportion of differences in depressive symptoms due to different occupations. In a next step, all individual-level factors are added to the analysis, resulting in a random intercept-model. The final model additionally contains individual and occupational-level covariates. As an indicator for model-fit, the Akaike information criterion (AIC) is reported for each model, with smaller values indicating better model-fit.

## Results

Table 1 provides a description of independent and dependent variables. The final sample consisted of 856/39.6% men and 1308/60.4% women with a mean age of 49.4 (SD: 8.1) and 49.3 years (SD: 7.7) for men and women, respectively, (nested in 65 distinct occupations. Each occupation included, on average, 33 workers (minimum: 10, maximum: 252). Mean CES-D-scores were 8.6 (SD: 5.4) for men and 10.8 (SD: 7.6) for women, respectively (overall score: 10.0, SD: 6.9). Women in our sample had higher values in neuroticism (mean = 3.4 vs. 3.0 in men, *P* < 0.001) and extraversion (3.8 vs. 3.6 in men; *P* < 0.001). Women reported slightly higher levels of social support (mean score: 17.5 vs. 17.3 in men, respectively, *p* = 0.38), however, differences were not significant. Only 1.85% of respondents reported a low level of education. More women than men had a middle level of education (67.8 vs. 52.2%), while men more often belonged to the highest education-category (40.4 vs. 30.7% in women; *P* < 0.001). No age differences were found in our sample.

**Table 1:** Sample description (overall/by gender)

	Total (*n* = 2164)		Men (*n* = 856)		Women (*n* = 1308)		*P*-value*
Variable	mean/%	SD	mean/%	SD	mean/%	SD	
Age	49.3	7.9	49.4	8.1	49.3	7.7	0.924
Education low (%)	1.9		2.3		1.5		*P* < 0.001
Education middle (%)	63.6		57.2		67.8		
Education high (%)	34.5		40.4		30.7		
Social resources	17.5	5.0	17.3	5.1	17.5	4.9	0.385
Neuroticism	3.2	1.1	3.0	1.1	3.4	1.2	*P* < 0.001
Extraversion	3.7	1.3	3.6	1.2	3.8	1.3	0.004
Interpersonal conflict	2.8	0.4	2.8	0.4	2.9	0.5	*P* < 0.001
Frequency. of conflictual contact	3.3	0.5	3.3	0.4	3.3	0.5	0.197
Dealing with physically aggressive people	1.8	0.5	1.7	0.5	1.8	0.5	*P* < 0.001
Dealing with angry people	3.4	0.5	3.3	0.5	3.5	0.5	*P* < 0.001
Depressive symptoms (CES-D-score)	10.0	6.9	8.6	5.4	10.8	7.6	*P* < 0.001

n = 2164; age reported in years; education assessed according to CASMIN (Comparative Analysis of Social Mobility in Industrial Nations)-classification categories low, middle, and high; neuroticism and extraversion assessed by the NEO-16-AM; social resources assessed by the Lubben Social Network Scale; CES-D = Center for Epidemiological Studies Depression Scale; * *p*-values based on Chi^2^- and two-sample t-tests, as appropriate

Women had slightly more contact with physically aggressive or angry people and higher overall-values for interpersonal conflict. No gender differences were found with regard to frequency of conflictual contact.

To illustrate different amounts of interpersonal conflict in specific occupations, examples of jobs with highest/lowest values in the respective types of contact are given in Table 2.

**Table 2:** Examples of occupations with high/low values of interpersonal conflict

Job variable	Highest values	Lowest values
*Dealing with angry people*	Policemen, educators, teachers, cashiers, nurses	IT-engineers, research fellows, manufacturing, architects, office/administrative staff, cosmeticians/hairdressers
*Dealing with physically aggressive people*	Policemen, tram/public transport drivers, (geriatric) nurses, teachers	Engineers, IT-sector, carpenters, architects
*Frequency of conflict situation*	(Geriatric) nurses, educators, teachers, lawyers, sales representatives	Lab assistants, kitchen aids, carpenters, office/administrative staff, commercial employees

Examples of jobs with lowest/highest values of interpersonal conflict on the job (2nd / 5th quintile, respectively, due to exclusion of lowest quintile)

Interpersonal conflicts were especially common in occupations entailing care work, e.g. nurses/hospital staff, but also jobs in the education or service sector. Lowest values were found in various types of office/administrative jobs and engineering professions.

The results of an overall-multilevel linear regression model with gender as a covariate are presented in Table 3. The likelihood-ratio-test confirmed the superiority of the multilevel model over OLS-regression (*P* < 0.001). Only a small proportion of variance (2.8%) in depressive symptomatology could be explained by differences between occupations, as indicated by the intraclass correlation coefficient (ICC, null model). This implies that variation in depressive symptomatology is for the most part due to differences between individuals, with a small level of variation explained by differences between occupations.

**Table 3:** Results of multilevel linear regression to assess depressive symptomatology (CES-D), total sample (n = 2164)

Variable	Null model		Model 1		Model 2	
	coeff.	95% CI	coeff.	95% CI	coeff.	95% CI
Individual level						
Intercept/constant	9.84	9.51; 10.14	7.53	4.70; 10.35	7.14	4.86; 10.04
Female gender			**1.20**	0.66; 1.74	**1.19**	0.56; 1.46
Age			0.00	−0.04; 0.03	0.00	−0.03; 0.02
Education: middle			−1.84	−3.68; 0.01	−1.84	−3.21; 0.01
Education: high (ref: low)			**−2.61**	−4.49; −0.72	**−2.60**	−4.04;-1.12
Social resources			**−0.23**	−0.28; − 0.18	**−0.23**	− 0.28; − 0.19
Neuroticism			**2.55**	2.33; 2.77	**2.55**	2.29; 2.67
Extraversion			**−0.37**	−0.56; − 0.17	**−0.37**	− 0.53; − 0.20
Occupational level						
Interpersonal conflict					0.14	−0.60; 0.83
Random effects						
Intercept SD	1.14	0.57; 1.28	0.51	0.24; 1.03	0.50	0.18; 0.86
ICC	0.028		0.008		0.007	
Log Likelihood	− 7233.77		− 6888.12		−6888.041	
LR-Test	Chi^2^ = 23.30; *P* < 0.001		Chi^2^ = 4.42; *P* = 0.018		Chi^2^ = 4.23; *P* = 0.020	
AIC	14,473.54		13,796.23		13,798.08	

*CI* confidence interval; *SD* standard deviation; education assessed according to CASMIN (Comparative Analysis of Social Mobility in Industrial Nations)-classification categories low, middle, and high; neuroticism and extraversion assessed by the NEO-16-AM; social resources assessed by the Lubben Social Network Scale; *CES-D* Center for Epidemiological Studies Depression Scale; significant associations presented in bold type

Women had higher levels of depressive symptoms than men (b = 1.2; 95% CI: 0.66–1.74; Model 1). Depressive symptoms were reduced in people reporting higher levels of social resources (b = − 0.23; 95% CI: − 0.28; − 0.18). Neuroticism was associated with increased depressive symptomatology, while higher levels of extraversion were linked to reduced depressive symptoms. No age differences were found. Education was linked to reduced risk of depressive symptoms, however, only for the highest level of educational attainment. Variation between jobs, indicated by the standard deviation of the random intercept, decreased from 1.14 to 0.51, indicating little variation of depressive symptoms between jobs.

In Model 2, we investigated associations between occupational-level covariates and depressive symptoms. The amount of conflictual contact was not linked to depressive symptoms (b = 0.19; 95% -0.31; 0.68). The intercept was 7.45 (95% CI: 4.86; 10.04). Associations with individual-level variables remained unchanged. Given the slightly smaller AIC-value, Model 1 should be considered the better-fitting model, i.e. the inclusion of interpersonal conflict does not improve the explanatory power of the model.

Tables 4 and Table 5 report the results of separate regression models for men and women. In the male subsample, 0.8% of variation in depressive symptoms could be explained by differences between occupations (null model). Multilevel regression was not superior to OLS regression, as indicated by the likelihood ratio test (*p* < 1.00). When entering individual-level factors (Model 1), social resources (b = − 0.2, 95% CI: − 0.26; − 0.13) and neuroticism (b = 1.86; 95% CI: 1.56; 2.16) were associated with depressive symptoms. Interpersonal conflict at work did not explain differences in depressive symptomatology (Model 2; = − 0.14; 95% CI: − 0.98; 0.69).

**Table 4:** Results of multilevel linear regression to assess depressive symptomatology (CES-D) in men (n = 856)

Variable	Null model		Model 1		Model 2	
	coeff	95% CI	coeff	95% CI	coeff	95% CI
Individual level						
Intercept/constant	8.65	8.26; 9.05	7.91	4.52; 11.31	8.30	4.20; 12.20
Age			0.03	−0.01; 0.07	0.03	−0.01; 0.07
Education: middle			−1.57	−3.71; 0.56	−1.60	−3.71; 0.56
Education: high (ref: low)			−2.08	−4.25; 0.08	−2.09	−4.26; 0.07
Social resources			**−0.20**	−0.26; − 0.13	**−0.20**	− 0.27; − 0.13
Neuroticism			**1.86**	1.56; 2.17	**1.86**	1.55; 2.16
Extraversion			−0.26	−0.55; 0.02	−0.26	− 0.54; 0.02
Occupational level						
Interpersonal conflict					−0.14	−0.98; 0.69
Random effects						
Intercept SD	0.50	0.11; 2.23	1.67*10^−6^	4.67*10^−9^; 0.00	3.63*10^−7^	0.00; 0.00
ICC	0.008		1.24*10^−13^		5.81*10^−15^	
Log Likelihood	− 2657.96		− 2549.59		−2549.53	
LR-Test	Chi^2^ = 0.55; *P* = 0.229		Chi^2^ = 0.00; *P* = 1.00		Chi^2^ = 0.00; P = 1.00	
AIC	5321.93		5117.17		5119.06	

*CI* confidence interval; *SD* standard deviation; education assessed according to CASMIN (Comparative Analysis of Social Mobility in Industrial Nations)-classification categories low, middle, and high; neuroticism and extraversion assessed by the NEO-16-AM; social resources assessed by the Lubben Social Network Scale; *CES-D* Center for Epidemiological Studies Depression Scale; significant associations presented in bold type

**Table 5:** Results of multilevel linear regression to assess depressive symptomatology (CES-D) in women (n = 1308)

Variable	Null model		Model 1		Model 2	
	coeff.	95% CI	coeff.	95% CI	coeff.	95% CI
Individual level						
Intercept/constant	10.75	10.18; 11.33	10.59	6.46; 14.72	10.06	4.96; 15.16
Age			−0.03	−0.07; 0.02	−0.03	−0.07; 0.02
Education: middle			−2.23	−5.07; 0.62	−2.21	−5.06; 0.63
Education: high (ref: low)			**−3.34**	−6.25; −0.43	**−3.33**	−6.24;-0.42
Social resources			**−0.27**	−0.34; − 0.19	**−0.27**	− 0.34; − 0.19
Neuroticism			**2.93**	2.63; 3.24	**2.94**	2.63; 3.24
Extraversion			**−0.32**	−0.59; − 0.06	**−0.32**	− 0.59; − 0.06
Occupational level						
Interpersonal conflict					0.17	−0.80; 1.14
Random effects						
Intercept SD	1.05	0.53; 2.08	0.65	0.28; 1.49	0.63	0.27; 1.50
ICC	0.019		0.010		0.001	
Log Likelihood	− 4500.89		− 4280.40		−4280.34	
LR-Test	Chi^2^ = 6.87; *P* = 0.004		Chi^2^ = 3.40; *P* = 0.032		Chi^2^ = 3.05; *P* = 0.040	
AIC	9007.77		8578.80		8580.68	

*CI* confidence interval; *SD* standard deviation; education assessed according to CASMIN (Comparative Analysis of Social Mobility in Industrial Nations)-classification categories low, middle, and high; neuroticism and extraversion assessed by the NEO-16-AM; social resources assessed by the Lubben Social Network Scale; *CES-D* Center for Epidemiological Studies Depression Scale; significant associations presented in bold type

Differences between occupations explained 1.9% of differences in depressive symptomatology in women (Table 5, null model). High levels of education and social resources were linked to lower levels of depressive symptoms (Model 1; b = − 3.34; 95% CI: − 6.25; − 0.34 and − 0.27; 95% CI: − 0.34; − 0.19, respectively). Neuroticism was associated with more, extraversion with less depressive symptoms. When entering occupational-level covariates, the regression coefficients did not change, interpersonal conflict was not associated with depressive symptoms in women (b = 0.17; 95% CI: − 0.80; 1.14). However, the likelihood ratio test indicated the superiority of a multilevel approach over OLS regression for the female subsample. In both subsamples, adding information on interpersonal conflict did not improve the quality of the model, as indicated by the AIC favoring Model 1 both for men and women.

## Discussion

Social conflict at work, as an objective job characteristic, was not associated with depressive symptomatology across 65 occupations in a large population-based sample. Differences in level of depressive symptoms were mainly explained by individual-level factors. The results do not confirm our hypothesis that social conflict at work is associated with higher levels of depressive symptoms. Some possible explanations for these findings are discussed below.

A possible interpretation is that job titles alone are too imprecise as indicators to be used in studies on occupational mental health: Jobs within the same occupation can vary largely between organizations, employers etc. regarding social relationships or the amount of conflict experienced [29, 65]. Regarding the small amount of variance due to occupational titles, more precise definitions of jobs or restriction to specific occupations might prove useful [38]. Conflicts at work were especially common among nurses, teachers or other professions in the service sector in our sample, corroborating existing evidence [23–26].

Since our analyses relied on cross-sectional data, we cannot rule out a possible selection bias/healthy-worker-effect, i.e. people with impaired mental health are probably less likely to work in occupations characterized by high levels of conflict.

Another possible explanation for why we did not find an association between work-related conflict and depressive symptoms points towards the assessment of interpersonal conflict in our study: While the majority of studies on occupational mental health uses subjective measures to assess work-related relationships or stressors, we relied on an objective measure, using a database including detailed evaluations of various aspects of work. The objective assessment of interpersonal conflict, however, might have contributed to the non-significant association. It is possible that an association would have been detected if subjective measures of job features had been used. Subjective assessments include individual perceptions of stressors such as interpersonal conflict at work, which might mediate the influence of occupational stressors [16, 41]. In other words: Objective job characteristics may be similar for all incumbents of an occupation, subjective perceptions and coping styles are not [66]. Expert ratings or average values of job characteristics for specific groups of workers might serve as more objective measures of workplace factors, but they might also capture less information about actual differences between individual working conditions [33]. This line of interpretation is supported by similar findings from the Whitehall II-study: Self-report measures of job strain were linked to depressive symptoms, whereas objective indices of job strain (i.e. expert ratings) were not [37]. A comprehensive review by van der Doef and Maes assessed studies testing the demand-control-(support) model and possible associations with mental health [67]. While overall there was much support for an association between job strain and impaired mental health when self-report measures were used, none of the studies in which job characteristics were assessed independently of the outcome measure supported a link with depressive symptoms. In a German study based on pension insurance data, conflicts in the workplace were found to be associated with higher likelihood of a depression diagnosis both in men and women [36]. However, this sample included only workers with a rehabilitation diagnosis, therefore excluding healthy cases and those with sub-clinical depressive symptoms. Comparisons between these findings and our study should be made with caution.

Individual-level covariates in our study mostly showed the expected association with depressive symptoms. Higher levels of neuroticism were associated with higher levels of depressive symptoms, higher levels of extraversion were linked to less depressive symptoms but only in women. Women reported more depressive symptoms than men, corroborating existing gender differences in the prevalence of depressive symptoms [68]. Men and women with higher levels of social resources reported less depressive symptoms. Social resources might be protective for mental health in general or ameliorate the impact of stressful events, e.g. from conflictual experiences in the workplace. This finding is in line with previous studies reporting a buffering effect of social resources on the impact of work-related stress [69, 70]. Higher levels of education were linked to less depressive symptoms, but only in women. Education can be understood as a form of personal capital or resource, enabling people to succeed e.g. in working contexts and to pursue personal goals [50]. Moreover, education can impact mental health indirectly since it generally enables access to higher-level jobs and higher income. This, however, was true only for the highest level of education and only for the female subsample, implying that education is protective against depressive symptomatology only beyond a certain threshold. Overall, our model was more appropriate for the female than for the male subsample, as indicated by likelihood ratio-tests and ICC. This might be due to less variation between occupations in the male subsample, leading to less explanatory power of the model.

### Strengths and limitations

One strength of our study is the use of objective measures of job qualities as included in the O*NET, providing a valuable measure of occupational characteristics. Since the information on work characteristics in the O*NET are assessed by incumbents and job experts, respectively, it provides a valuable measure of interpersonal relationships in different occupations that is meaningful to interpret. Many studies in occupational health psychology share the common problem that specific instruments or questionnaires on job stressors are more adequate for certain occupations than for others [38], a risk that can – at least partially – be avoided when using information from the O*NET database. Comparable studies are rare in Germany and similar databases for the German workforce are not available so far. We used a large, population-based sample including a wide range of occupations, making the sample less selective than those in many previous studies. It has been pointed out that of the vast variety of occupations, only few have been studied in detail regarding their associations with mental health and depression [71].

We chose a multilevel framework for our research question based on theoretical grounds, since the qualities we wanted to investigate were assessed as features of jobs rather than of people. As a more technical indicator, the likelihood ratio test confirmed the nested structure of the data, indicating the superiority of a multilevel approach over OLS regression. A growing body of literature in the field of occupational mental health confirms this approach, reporting, on average, smaller associations than those found in OLS regressions and little variation between occupations [14, 29, 37, 40].

We restricted our analysis sample to people who experience at least a certain amount of interpersonal conflict at work by excluding the lowest quintile of values for the respective job characteristics. This might make our results more robust against statistical outliers and give a more accurate impression of the association between interpersonal conflict and depressive symptoms.

Certain limitations need to be addressed when interpreting our findings. First, since our study relies on cross-sectional data, no conclusions about causality can be drawn. Unfortunately, some potentially valuable information was not included in the LIFE-Adult baseline assessment, for example on the duration the subjects had been employed in the respective occupations. It might be possible that e.g. long periods of working in an occupation with high levels of interpersonal conflict may indeed raise the risk for depression. Then again, job experiences and histories of employment might provide useful resources and coping strategies which could possibly protect against work-related stressors. These questions, however, cannot be answered within the current study. Moreover, our data did not contain information on job involvement or employees’ motivation as a potential moderating factor. However, comparable studies found little [72] or no support [73, 74] for job involvement having any influence on depressive symptoms or other mental health outcomes, therefore, the impact of this factor seems negligible.

Unfortunately, a substantial part of the original sample was lost due to missing values in the explanatory variables. However, a non-responder analysis revealed no differences in depressive symptomatology between responders and non-responders; therefore, this should not have influenced the results in a substantial way.

Lastly, a possible limitation arises from applying occupational information from a US-American database to a study sample from Germany. Since the O*NET data refer to the US-American labor market, slight differences regarding e.g. responsibilities, work context and social contacts made at work might occur between the same occupations in the US and Germany, respectively. However, since the O*NET data have previously been used in other health-related studies conducted in Germany [36, 75, 76], these possible differences should be negligible.

## Conclusions

This study contributes to the literature on occupational factors and depressive symptoms using multilevel analyses. As in many comparable studies investigating the link between certain job aspects and mental health using individual- and job-level information, the association is not significant and job-level factors account for only little variance in depressive symptomatology. Our findings suggest that the association of interpersonal conflict at work and depressive symptoms does not differ between occupations. It can be assumed that approaches focusing only on the individual level of analysis via e.g. self-report measures tend to report more and stronger associations with depressive symptoms. A reason for this might be that it is less the objective job feature than rather people’s individual *perception* of their job, i.e. cognitive and affective assessments of job characteristics, that are associated with depressive symptoms. If this was the case, strategies for prevention should especially focus on employees’ perceptions of their jobs, promotion of psychosocial resources and individual assessments of oneself and the workplace. Against this background, further discussions on the possible factors of jobs that are associated with depressive symptoms should put a stronger focus on methodological questions and possible ways of conceptualizing research questions. This could help to disentangle the pathways through which individual and job-related factors impact workers’ mental health.

The role of psychosocial aspects of employment and possible links to depression is still inconclusive. It can be argued that specific stressors in the workplace are more amendable to change than global frameworks like “work stress” or “job strain”, therefore, further research addressing aspects like social relations in the workplace is highly warranted. Future investigations should be more precise about questions of operationalization and methodology: Does the study assess features of jobs or rather subjective perceptions of occupational environments, the latter reflecting both the stressor and its evaluation by the incumbent? Further research taking into account occupations and/or organizations which employees are nested in can shed more light on the factors that pose a danger to mental health.

## Data Availability

The dataset analyzed during the current study is available from the corresponding author upon reasonable request.

## Abbreviations

AIC: Akaike information criterion
CASMIN: Comparative Analysis of Social Mobility in Industrial Nations
CES-D: Center for Epidemiological Studies Depression Scale
CI: Confidence interval
ICC: Intra-class correlation coefficient
LSNS: Lubben Social Network Scale
NEO-16 AM: NEO-16 Adjective Measure
O*NET: Occupational Information Network
OLS: Ordinary least squares
SD: Standard deviation
